# Attention-Based Automated Feature Extraction for Malware Analysis

**DOI:** 10.3390/s20102893

**Published:** 2020-05-20

**Authors:** Sunoh Choi, Jangseong Bae, Changki Lee, Youngsoo Kim, Jonghyun Kim

**Affiliations:** 1 Department of Computer Engineering, Honam University, Gwangju 62399, Korea; 2 Department of Computer Science and Engineering, Kangwon University, Kangwon-do 24341, Korea; jseffort88@gmail.com (J.B.); leeck@kangwon.ac.kr (C.L.); 3 Information Security Division, Electronics and Telecommunications Research Institute, Daejeon 34129, Korea; blitzkrieg@etri.re.kr (Y.K.); jhk@etri.re.kr (J.K.)

**Keywords:** malware analysis, deep learning, attention

## Abstract

Every day, hundreds of thousands of malicious files are created to exploit zero-day vulnerabilities. Existing pattern-based antivirus solutions face difficulties in coping with such a large number of new malicious files. To solve this problem, artificial intelligence (AI)-based malicious file detection methods have been proposed. However, even if we can detect malicious files with high accuracy using deep learning, it is difficult to identify why files are malicious. In this study, we propose a malicious file feature extraction method based on attention mechanism. First, by adapting the attention mechanism, we can identify application program interface (API) system calls that are more important than others for determining whether a file is malicious. Second, we confirm that this approach yields an accuracy that is approximately 12% and 5% higher than a conventional AI-based detection model using convolutional neural networks and skip-connected long short-term memory-based detection model, respectively.

## 1. Introduction

Every day, hundreds of thousands of malicious files are created to exploit zero-day vulnerabilities [1,2] For this reason, the existing pattern-based antivirus solutions face difficulties in responding to new malicious files [3]. Traditional pattern-based antivirus solutions determine whether files are malicious by evaluating their hash values, their string content, or their behavior. However, new malicious files are being designed to avoid detection by existing antivirus solutions. Therefore, malware detection requries an alternative solution.

Methods that use artifical intelligence (AI) for detecting malicious files have been recently proposed [3,4,5,6,7,8,9,10]. AI has been widely employed for image recognition and machine translation [11,12]. Machine learning and deep learning techniques are widely utilized in AI applications. An advantage of AI is that it can effectively identify data that are similar to the training data. That is, even if there are no patterns for a new malicious file, it is possible to determine whether it is malicious [13]. Kaspersky Internet Security that uses machine learning has improved accuracy.

If we add the AI-based malware detection method to the existing anti-virus software, we can increase the malware detection rate because we can detect malware that is not found by the existing anti-virus software. It is known that, when using machine learning, the detection rate is higher and the false positive rate is less than that of pattern-based detection [13]. When a new malware is exposed to pattern-based antivirus software, it cannot establish whether it is malicious because the pattern is not recognized. However, the AI-based malware detector determines whether it is malicious with high probability.

**Figure 1:**
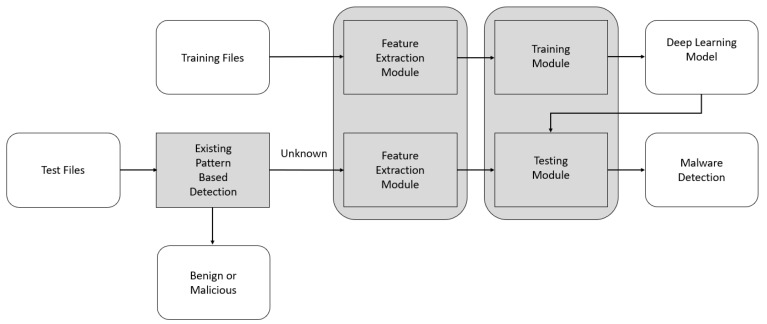
Malware detection system structure based on artificial intelligence.

To detect malicious files based on AI using a PC, two modules are generally required: a malicious file feature extraction module and an AI-based training and testing module, as illustrated in Figure 1. Note that we can also use the AI-based malware detection method in a server, on a smartphone, or in the cloud. However, the feature extraction modules would be slightly different according to the operating systems. Because portable executable (PE) files are executed on the Windows OS and Android application package (APK) files are executed on the Android OS, each requires a different feature extraction module. However, the deep learning models for malware detection are similar.

As depicted in Figure 1, when the training files are available, the features are extracted using a feature extraction module. Thereafter, once these features have been used to train the deep learning model in the training module in a server or PC, we obtain the trained deep learning model.

Next, when a test file is provided, the existing pattern-based antivirus software examines it. However, when it is new and the antivirus does not have the pattern in its database, it reports that it cannot establish whether it is malicious. Note that, if it has a pattern, it will determine whether it is malicious, but, if it cannot be decided, the suspect item is passed to the feature extraction module for AI-based malware detection.

We extract the relevant features using the feature extraction module and determine whether the file is malicious by using the trained deep learning model in the testing module in a user’s PC. Note that we run the training and testing modules in a PC for simplicity. However, the training module is run by a malware detection system administrator and the testing module is run by a user. In addition, even if the training time is relatively long, the testing time is short. Therefore, we can detect malware using the AI-based detection method at runtime.

To extract feature data from malicious files, we can use static [4,5,14,15,16,17] and dynamic analyses [6,7,8] Static analysis involves the extraction of features such as strings and opcodes, and dynamic analysis extracts features such as application program interface (API) system calls by executing the malware.

Thereafter, by using extracted features with an AI model such as a convolutional neural network (CNN)-based model or long short-term memory (LSTM)-based model, we can determine whether the file is malicious or not. However, in the AI model, we do not know why the file is malicious. Therefore, we must determine which features are more significant than others in a malicious file.

In this study, we propose a malicious file feature extraction method based on attention mechanisms [18]. An attention mechanism calculates the weight of each input value of the deep learning model, in order to determine which has a greater impact on the result. Thus, using this mechanism, we can determine which features are more significant than others. To the best of our knowledge, this study is the first to apply the attention mechanism to malicious file feature extraction. Furthermore, our experimental results indicate that this method increases the accuracy of the AI-based malicious file detection system and determines in reasonable time whether the file is malicious.

The remainder of this paper is organized as follows: Related research is discussed in Section 2. Basic malicious file feature extraction method and basic deep learning techniques for malicious file detection are presented in Section 3. A new method for malicious file feature extraction is detailed in Section 4. In Section 5, we demonstrate that the proposed method outperforms existing methods. Finally, we provide a discussion and conclusions in Section 6 and Section 7, respectively.

## 2. Related Work

Methods of analyzing malicious files can be categorized into static and dynamic analysis methods. Static analysis methods judge whether a file is malicious by analyzing strings [14], import tables [15], byte n-grams [16], and opcodes [16]. Static analysis methods can analyze malicious files relatively quickly, although they face difficulties analyzing files when they are obfuscated or packed [17]. With dynamic analysis methods, malicious files are analyzed by running them. However, these methods have the disadvantage that malicious files may detect the virtual environment and not operate in it [4]. It may also be difficult to collect complete malicious behavior because malicious files may only run in certain circumstances.

Recently, there have been many studies on malicious file analysis using AI. The methods in [4,5] utilize a static analysis to analyze malicious files. The method in [4] extracts byte histogram features, import features, string histogram features, and metadata features from a file and trains deep learning models based on deep neural networks. The method in [5] learns a convolutional neural network (CNN)-based deep learning model by extracting opcode sequences.

The approaches in [6,7,8] utilize a dynamic analysis method to analyze a malicious file, and execute it to collect the API system call and extract a subsequence of a certain length from the system call sequence using a random projection. The method in [6] utilizes a deep neural network-based deep learning model, and that in [7] employs a deep learning model based on a recurrent neural network (RNN). The authors of [8] proposed a deep learning model that simultaneously performs malicious file detection and malicious file classification. In the present study, we employ both static and dynamic analyses. Furthermore, a novel property is that we propose automatic feature extraction based on the attention mechanism.

Even if malicious files can be detected with high accuracy using deep learning, we do not know which API system calls are more important than others. Recently, the attention mechanism was proposed in the neural machine translation community to provide sentence summarization [18]. To our best knowledge, this study represents the first attempt to analyze and detect malicious files by adapting the attention mechanism. By utilizing the attention mechanism, we can determine which API system calls are more important in malicious files, and our approach yields a higher accuracy than existing malware detection models.

Here, we focus on malicious Portable Executable (PE) files, but other types of malicious files also exist. These include malicious PDF files [19] and PowerShell scripts [20,21]. Some researchers have attempted to determine whether PDF files and PowerShell scripts are malicious using AI. However, such approaches are outside the scope of the present study.

## 3. Deep Learning Based Malware Detection

Deep learning-based malware detection requires two modules. The first is feature extraction and the second is a deep learning model. In Section 3.1, we introduce two feature extraction methods. In Section 3.2, we present three deep learning models.

### 3.1. Feature Extraction for Malware Detection

In this section, we provide two feature extraction methods. The first is static analysis-based feature extraction and the second is dynamic analysis-based feature extraction.

#### 3.1.1. Feature Extraction Using Static Analysis

**Figure 2:**
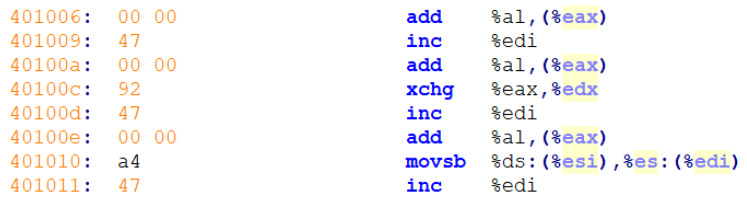
PE file assembly code.

Malicious file feature data are required for malicious file detection using deep learning. To this end, we can extract malicious file feature data using static or dynamic analyses. First, for a static analysis, we extract the assembly code using objdump [22], as shown in Figure 2. Next, we extract the opcode sequences such as add, inc, and add. We can then construct a trigram sequence [23] for three consecutive opcodes. The trigram sequence is created as follows:

(add, inc, add), (inc, add, xchg), (add, xchg, inc), …

The reason for creating trigram sequences is that there are approximately 100 opcodes, and when these are placed into trigrams, the size of the trigram domain is approximately $100^{3}$, such that the trigram sequence of each file can easily be distinguished from those of other files.

#### 3.1.2. Feature Extraction Using Dynamic Analysis

Next, we employ the Cuckoo Sandbox [24] to extract the API system calls of the portable executable (PE) file. An example of an API system call list is presented in Table 1. We first run the Cuckoo Sandbox, and then extract the API system call sequence. In addition, we extract the trigrams for three consecutive API system calls to create a trigram sequence.

### 3.2. DL-Based Malware Detection Model

In this section, we introduce three deep learning models for malware detection. The first is recurrent neural network, the second is LSTM, and the third is skip-connected LSTM (SC-LSTM).

#### 3.2.1. Recurrent Neural Network (RNN)

**Figure 3:**
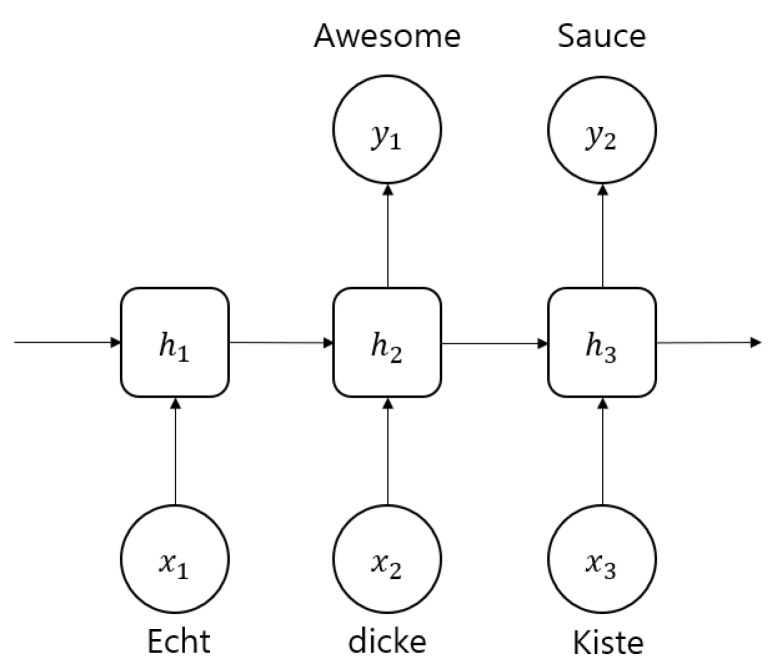
RNN for machine translation.

Here, we introduce a basic deep learning model used for malicious file detection. RNNs are mainly employed to process sequential information, such as language translation [25]. Figure 3 illustrates the structure of a typical RNN used to translate German sentences into English. However, RNNs have the disadvantage that the longer the input sentence, the smaller the influence of the preceding words. This is known as the vanishing gradient problem [26].

#### 3.2.2. Long Short Term Momory (LSTM)

**Figure 4:**
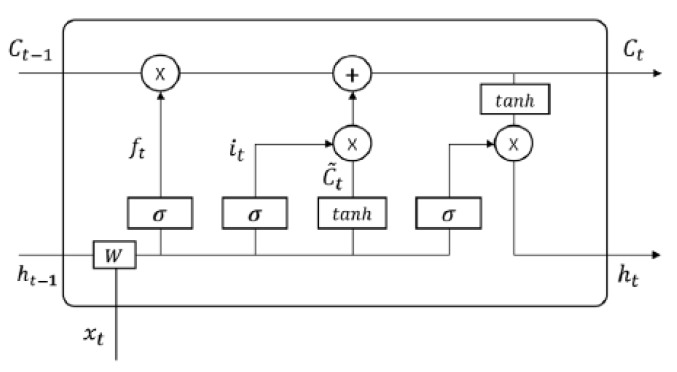
LSTM structure [27].

LSTM [27] was proposed to solve the aforementioned vanishing gradient problem [26]. LSTM has three gates, as shown in Figure 4. The gates are a way to optionally let information through. They are composed of a sigmoid neural net layer σ and a pointwise multiplication operaion ×. The first is the forget gate $f_{t}$, which determines whether the state $C_{t-1}$ of the previous cell is reflected as follows:

(1) $$f_{t}=\sigma \left(W_{f}\cdot \left[h_{t-1},x_{t}\right]+b_{f}\right)$$

where $x_{t}$ is an input, $h_{t-1}$ is the output of the previous cell, $W_{f}$ is a weight, and $b_{f}$ is a bias.

The second is the input gate $i_{t}$, which determines how much the state of the previous cell will be updated as follows:

(2) $$i_{t}=\sigma \left(W_{i}\cdot \left[h_{t-1},x_{t}\right]+b_{i}\right)$$

(3) $$\tilde{C_{t}}=tanh\left(W_{t}\cdot \left[h_{t-1},x_{t}\right]+b_{C}\right)$$

where $\tilde{C_{t}}$ is a new candidate value. Then, the state $C_{t}$ of the next cell is calculated as follows:

(4) $$C_{t}=f_{t}*C_{t-1}+i_{t}*\tilde{C_{t}}$$

The third is the output gate $o_{t}$. The output $h_{t}$ of the current cell is calculated as follows:

(5) $$o_{t}=\sigma \left(W_{o}\cdot \left[h_{t-1},x_{t}\right]+b_{0}\right)$$

(6) $$h_{t}=o_{t}⊗tanh\left(C_{t}\right)$$

In LSTM, the state $C_{t-1}$ of the previous cell has the possibility to be changed less than in an RNN. Therefore, LSTM has the advantage that the initial state of a cell can be better reflected.

#### 3.2.3. Skip-Connected LSTM

A residual network is a technology that solves the problem that learning and evaluation errors do not decrease in the image recognition field using a CNN model, even when the depth of the network is increased. This technique adds a hidden input layer followed by some steps to present inputs without weight calculations [11].

**Figure 5:**
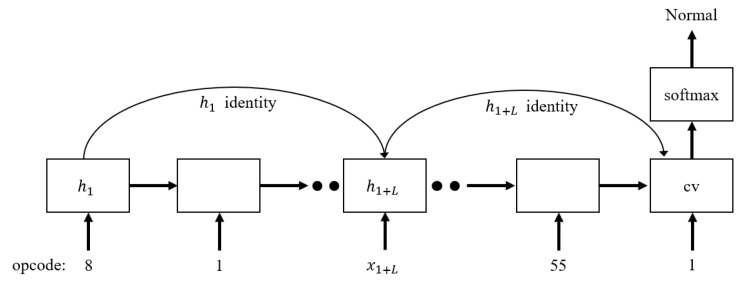
Skip-connected LSTM structure.

To improve the information flow of the LSTM network while maintaining the advantages of the gate mechanism, it has been proposed to introduce skip connections between two LSTM hidden states [12]. The result is called SC-LSTM. The structure of this model is illustrated in Figure 5.

SC-LSTM has the advantage that the dependencies of long sequence information are better captured by skip connections. The next hidden state is calculated as follows:

(7) $$h_{t+L}=o_{t+L}⊗tanh\left(C_{t+L}\right)+h_{t}$$

## 4. Automated Feature Extraction Based on Attention

In this section, we propose an automated feature extraction method based on the attention mechanism. First, in Section 4.1, we introduce the attention mechanism. Subsequently, in Section 4.2, we present an attention-based feature extraction method for malware detection.

### 4.1. Attention Mechanism

**Figure 6:**
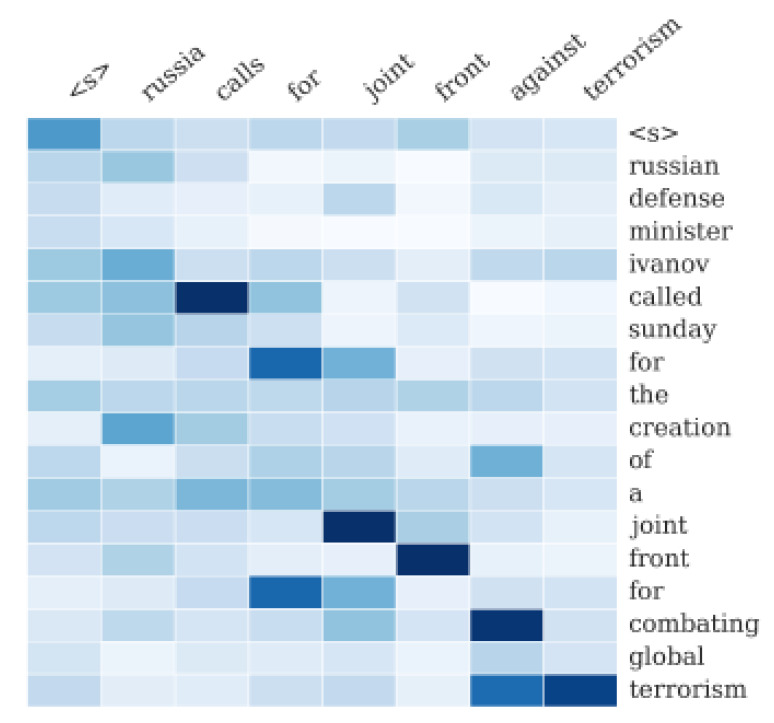
Alignment of text summarization [18].

Attention is a deep learning mechanism that looks for the parts of sequence data with greater impacts on the results. A typical example of attention is text summarization [18] that involves summarizing a given text. Using the attention mechanism, we can identify some of the main words to summarize an article when it is given as a sequence of words. For example, the text shown in Figure 6 is summarized as follows:

(8) russiacallsforjointfrontagainstterrorism

The RNN model is utilized in neural machine translation (NMT). For example, German sentences can be translated into English. NMT encodes the source sentence into a vector and decodes the sentence based on that vector.

**Figure 7:**
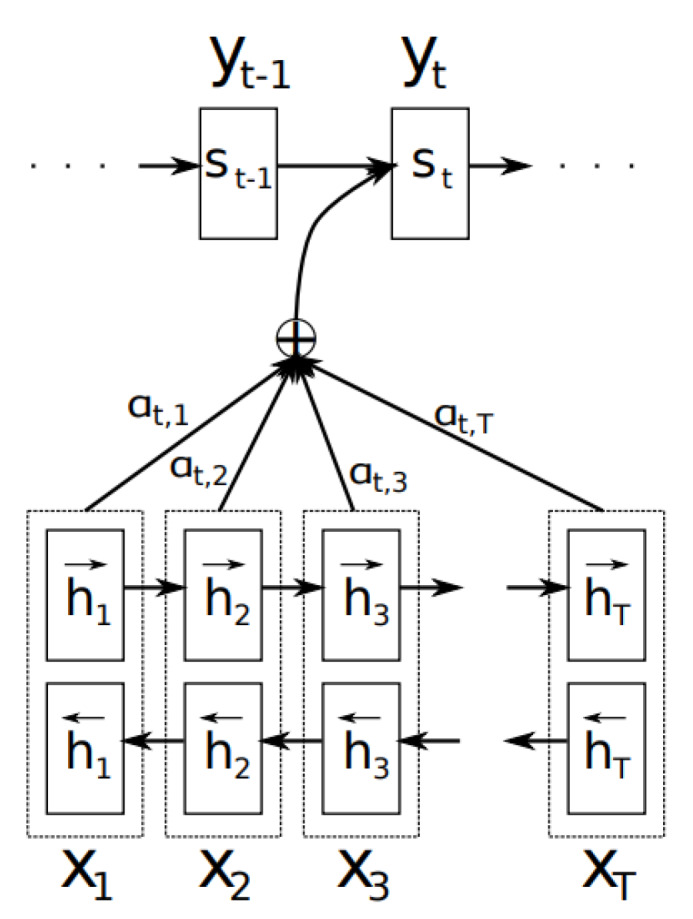
Attention [25].

The attention mechanism allows the decoder to refer to a portion of the source sentence [25], as shown in Figure 7 [25]. Here, *X* is the source sentence and *y* is the translated sentence generated by the decoder. Figure 7 depicts a bidirectional RNN. The important point is that the output word $y_{t}$ depends on the weight combination of all input states. Here, α is a weight that defines how strongly each input state influences each output state. If α(3,2) is large, it means that the third word in the output sentence refers to the second word in the input sentence.

In the attention model, the hidden state $S_{t}$ is defined as follows:

(9) $$s_{t}=f\left(s_{t-1},y_{t-1},c_{t}\right)$$

Furthermore, the context vector $c_{t}$ is defined as follows:

(10) $$c_{t}=\sum_{j=1}^{T}\alpha_{i,j}X_{j}$$

Weight $\alpha_{i,j}$ is computed as

(11) $$\alpha_{i,j}=\frac{exp(e_{i,j})}{\sum_{k=1}^{T}exp\left(e_{i,k}\right)}$$

In addition, $e_{i,j}$ is computed as follows:

(12) $$e_{i,j}=a\left(s_{i-1},X_{j}\right)$$

Here, $e_{i,j}$ is an alignment model indicating how well the input *j* and output *i* are matched.

The advantage of attention is that it provides the ability to interpret and visualize what the model does. For example, by visualizing the attention weight matrix when a sentence is translated, we can understand how the model performs translation. As shown in the text summarization above, we can observe how strongly each word in the output summary statement refers to each word in the input sentence.

### 4.2. Feature Extraction Based on Attention Mechanism

In this section, we propose an automatic feature extraction method based on the attention mechanism. The idea behind this method is as follows: When we utilize the attention model to identify the weight of each API system call in a sequence of length *n*, we find that the malicious file detection rate increases when we consider subsequences of length *k* by extracting weighted words for malicious file detection.

For example, the API sequence for a malicious file might may appear as follows:

{LoadLibrary,LoadCursor,RegisterClass,GetThreadLocal,Strcmp,

GlobalAlloc,GlobalFree,FindResource,LoadResource,VirtualProtect}

In the attention model, the weights of this sequence may be expressed as follows:

{0.3,0.0125,0.0125,0.0125,0.3,0.0125,0.0125,0.0125,0.0125,0.3}

Here, we extract the APIs with the top three weights:

{LoadLibrary,strcmp,VirtualProtect}

This is the API sequence pattern that appears in import address table-hooking malicious files [9]. We expect the detection rate of malicious files to increase using subsequence data with the top three weights through the attention model, rather than using the full sequence of length 10.

The purpose of the automatic feature extraction method is as follows: First, significant subsequences of length *k* are extracted from a data sequence of length *n* extracted from a malicious file, to enhance malicious file detection performance through deep learning.

After extracting significant data subsequences, malicious file analysts can analyze malicious files more easily. That is, malicious file analysts can analyze malicious files by analyzing significant subsequences of data, rather than examining the entire data sequence.

**Figure 8:**
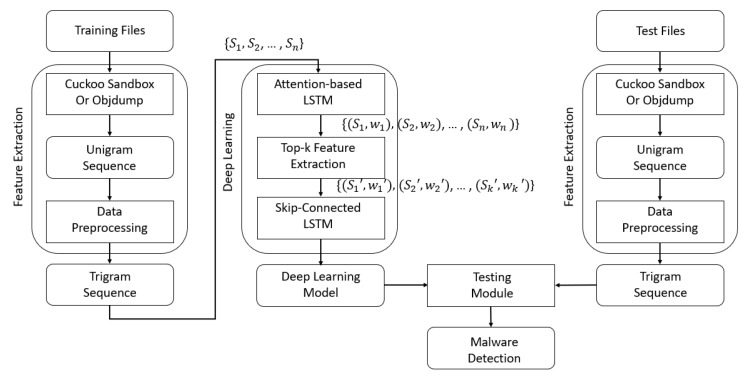
Structure of the automatic feature extraction module.

The automatic feature extraction method based on the attention mechanism is described as follows: First, when files are provided, we extract features such as API system call sequences using Cuckoo Sandbox. Note that we can extract features such as opcode sequences by static analysis instead of dynamic analyses such as Cuckoo Sandbox. Second, we construct a trigram sequence $\left\{S_{1},S_{2},\cdots ,S_{n}\right\}$ for three consecutive data. Third, we train the LSTM model based on the attention mechanism, and calculate the weight $W_{i}$ of each data point $S_{i}$ in the data sequence $\left\{S_{1},S_{2},\cdots ,S_{n}\right\}$, as depicted in Figure 8:

(13) $$\left\{\left(S_{1},W_{1}\right),\left(S_{2},W_{2}\right),\cdots ,\left(S_{n},W_{n}\right)\right\}$$

Next, we extract the data subsequence with the *k* highest weights, as follows:

(14) $$\left\{\left(S_{1}^{\prime },W_{1}^{\prime }\right),\left(S_{2}^{\prime },W_{2}^{\prime }\right),\cdots ,\left(S_{k}^{\prime },W_{k}^{\prime }\right)\right\}$$

Finally, this subsequence is utilized as data for the deep learning model for malicious file detection. As depicted in Figure 8, we obtained the trained deep learning model using the subsequence. When a test file is given, we can determine whether the file is malicious in the testing module using the trained deep learning model. We demonstrate our proposed method in the next section.

## 5. Experimental Results

### 5.1. Setup

All experiments were performed on the Ubuntu operating system, and the models used in the experiments were implemented using Theano [28]. The detailed experimental conditions are described in Table 2.

### 5.2. Data

The files utilized in the experiments were PE files collected from HAURI [29]. We used 1000 normal and 1000 malicious files. Table 3 presents the types of malware: trojan, win32, backdoor, worm, dropper, malware, and virus. These were classified using Ahnlab V3 [30]. Note that the purpose of this study is only to determine whether the files are malicious and not to classify the type of malware. Different malware families may produce different detection results. There are other studies on malware classification [31], but this is beyond the scope of this research. However, we will consider the topic in future work.

**Figure 9:**
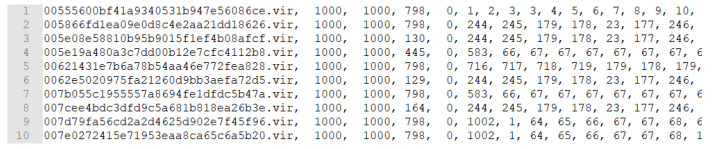
Trigram sequence of normal files.

**Figure 10:**
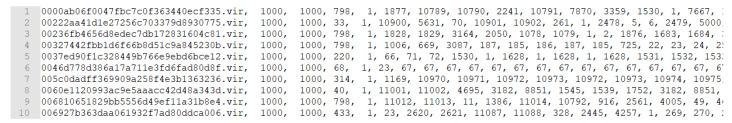
Trigram sequence of malicious files.

We extracted the API system call sequences from these PE files using the Cuckoo Sandbox. These consisted of API unigram sequences. We then converted the three consecutive API system calls into one trigram to create API trigram sequences, as depicted in Figure 9 and Figure 10. Note that some PE files did not execute well in the Cuckoo Sandbox; therefore, we removed the API sequences of length less than 5. Finally, we used 690 normal files and 785 malicious files in the experiments. Each line in Figure 9 and Figure 10 provide the following information:

{File Name, PID (dummy), PPID (dummy), sequence length,

Normal (0) or Malicious (1), API Trigram Sequence}

Note that the length of the API unigram sequence was set to 800. Because we converted the three consecutive API system calls into one API trigram, the length of the API trigram sequence is 798. When the length is less than 798, it is padded with zeros.

**Figure 11:**
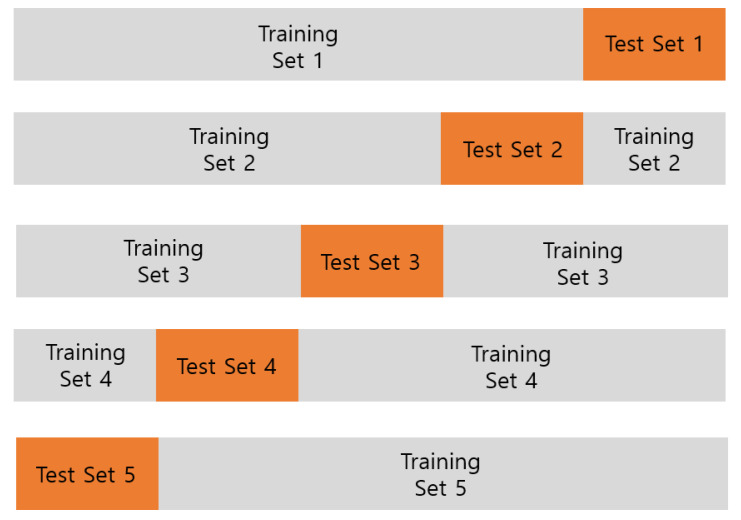
Five-fold cross-validation.

Subsequently, we used five-fold cross validation [32] as depicted in Figure 11. Cross-validation is a statistical method used to estimate the skill of deep learning models. We randomly divided the dataset into five subsets. In the first experiment, the first four subsets were used for the training and the fifth subset was used for the test. In the fifth experiment, the first subset was used for the test and the other four subsets were used for the training.

The first experiment was performed to demonstrate the effect of attention. To this end, we compared the accuracy of the attention-based model with those of the CNN-based [5] and SC-LSTM-based models [10]. Note that we implemented the CNN-based model using Keras [33] instead of Theano [28]. The second experiment was conducted to measure the time required to train and test the attention-based model which was compared with the time required to train and test the CNN-based and SC-LSTM-based models.

### 5.3. Performance Metric

In this section, performance evaluation indicators are described, before presenting the experimental results. The indicators utilized in this study are accuracy, true positive rate (TPR), and false positive rate (FPR). The confusion matrix used to calculate these values is shown in Table 4.

TP indicates that a file has been correctly evaluated by the system to be malicious, and TN indicates that the system correctly determined that a benign file is normal. Furthermore, FP indicates that a normal file has been incorrectly judged by the system as malicious, and FN indicates that the system has incorrectly identified a malicious file to be normal. Each indicator is calculated as follows:

(15) Accuracy=(TP+TN)/(TP+FP+FN+TN)

(16) TPR=TP/(TP+FN)

(17) FPR=FP/(FP+TN)

Accuracy refers to the rate at which the system correctly determines both malicious and normal files. The higher the value, the better the performance. TPR refers to the rate at which the system correctly identifies malicious files as malicious. The higher the value, the better the detection performance. FPR refers to the rate at which the system identifies normal files as malicious, and the lower the value, the better the detection performance. All experiments were performed using 5-fold cross validation.

### 5.4. Results

#### 5.4.1. Accuracy

**Figure 12:**
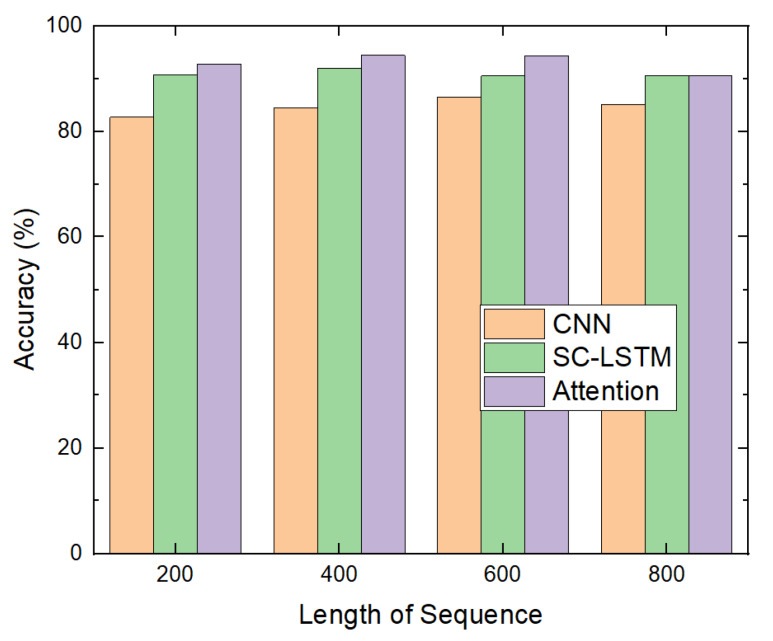
Accuracy.

The first experiment was conducted to demonstrate the effect of the attention mechanism for malware detection. The accuracy of the attention-based detection model was compared with those of the CNN-based and SC-LSTM-based models, as shown in Figure 12. The attention-based model yielded an accuracy that was approximately 12% and 5% higher than those of the CNN-based and SC-LSTM-based models, respectively. Table 5 shows the numbers of true positives, false positives, false negatives, and true negatives for each model.

Note that, when the length of the sequence was 800, the accuracy of the attention-based model was the same as that of the SC-LSTM-based model because the length of the original sequence was limited to 800. This indicated that, when the length was 800, the input sequence of the attention-based model was the same as that of the SC-LSTM-based model. In this case, the attention-based model did not have any effect. When the length of the sequence was less than 800, the attention-based model extracted subsequences whose API system calls were more important. Thus, the attention-based model yielded a higher accuracy than the others.

#### 5.4.2. Training Time

**Figure 13:**
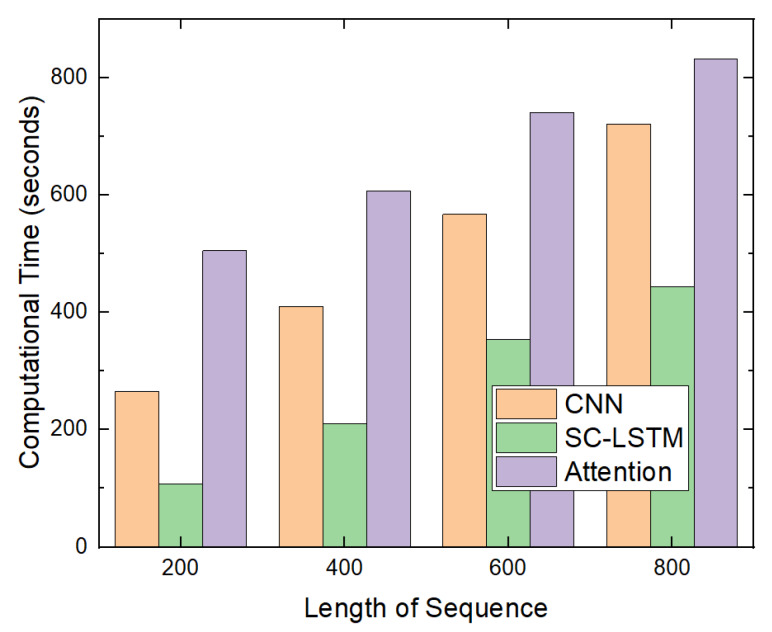
Training time.

The second experiment measured the computational time of the attention-based detection model. This was compared with the computational times of the CNN-based and SC-LSTM-based models, as shown in Figure 13. The computational time of the attention-based model consisted of the training time of the attention-based model, whose input sequence length was 800, time required to extract the top-k API system calls, and time needed to train the SC-LSTM-based model whose input sequence length was k.

Note that, in the attention-based detection model, we first computed the weights of each API system call in the input sequence. Then, we extracted the top-k API system calls and created a subsequence. Next, we trained the SC-LSTM-model using the extracted subsequence.

Because we implemented the CNN-based model using Keras [33] instead of Theano [28], the computational time for the CNN-based model was longer than that for the SC-LSTM-based model. Training the attention-based model using an input sequence of length 800 required approximately 400 s. Therefore, the computational time of the attention-based detection model was longer than that of the SC-LSTM-based model.

However, the attention-based detection model yielded a higher accuracy. In addition, by using the attention-based model, we can identify which API system calls are more important to determine whether a file is malicious.

#### 5.4.3. Test Time

The third experiment measured the time required to test the attention-based detection model using a GPU. Once the system call information is available, the time taken to establish whether the 280 test files are malicious in the deep learning model can be determined. Note that the test time does not include the time required to extract the API system call sequence using Cuckoo Sandbox. To extract the API system call sequence using Cuckoo Sandbox, we executed each file for 3 min. However, modern anti-virus software contains a module to extract API system calls in real time [30]. Therefore, we believe that malware can be detected using the attention-based detection model in real time.

**Figure 14:**
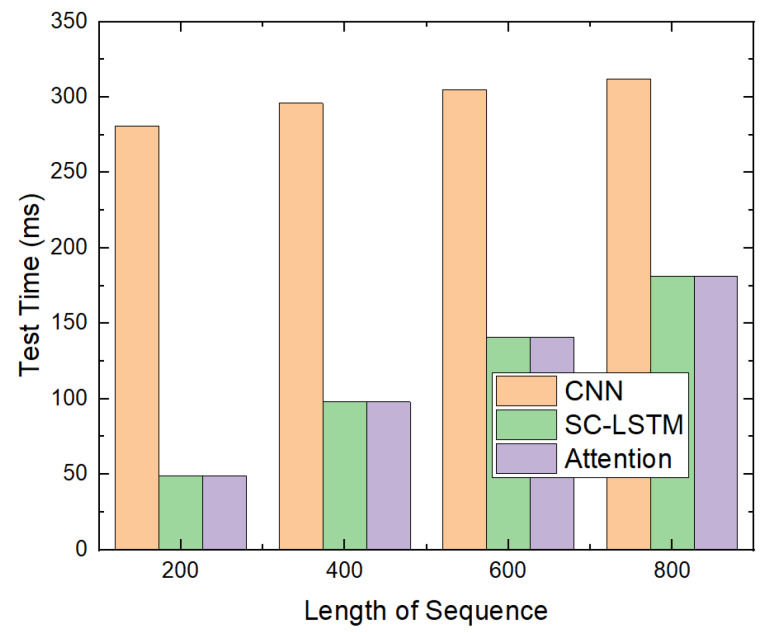
Time required to test using GPU.

The time to test the attention-based detection model was compared with what is required to test the CNN-based and SC-LSTM-based models, as depicted in Figure 14. The time required to test the attention-based model is the time required to determine whether the file is malicious. The number of test files was 280.

Because we implemented the CNN-based model using Keras [33] instead of Theano [28], the test time for the CNN-based model was longer than that for the SC-LSTM-based model. Because the attention-based model uses the same sized neural network as the SC-LSTM-based model, the test time for the attention-based model was the same as that for the SC-LSTM-based model, and it was proportional to the length of the API trigram sequence. When there were 280 test files and the length of the API trigram sequence was 800, it required approximately 180 ms. We believe that this is reasonable.

**Figure 15:**
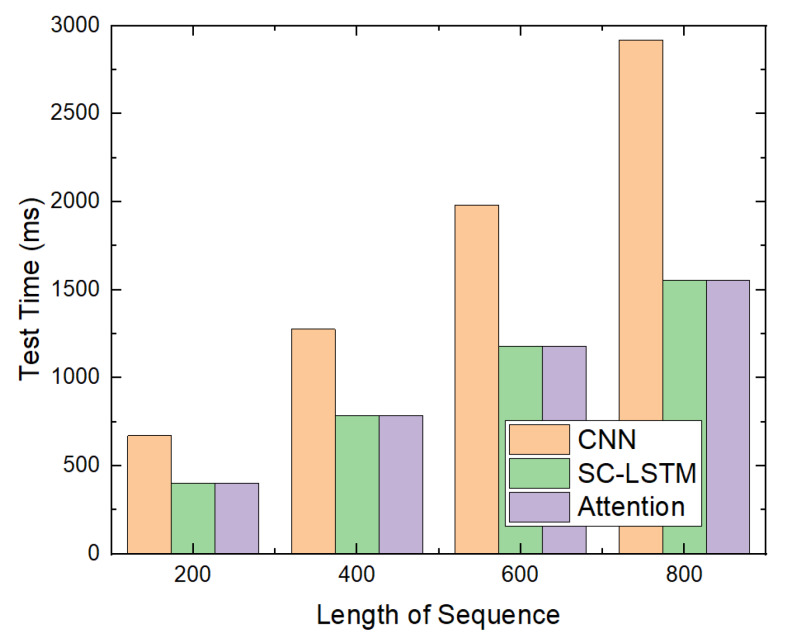
Time required to test only using CPU.

In addition, we measured the time required to test the attention-based detection model using only the CPU. This was compared with the time required to test the CNN-based and SC-LSTM-based models using only the CPU, as presented in Figure 15. The time required to test the attention-based model is the time required to determine whether the file is malicious using only the CPU.

When only the CPU was used, the time required to test the attention-based detection model was significantly longer than when the GPU was used. This implies that we can reduce the time required to test by using the GPU because it supports parallel computing in the deep learning model. Moreover, when using only the CPU, the time required to test the CNN-based model is proportional to the length of the sequence. It appears that, because the CPU does not support parallel computing in the deep learning model, the test takes a significantly longer time.

**Figure 16:**
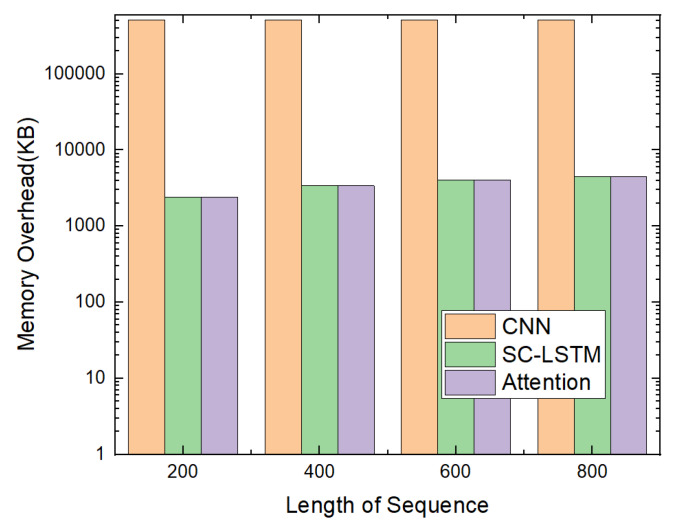
Memory overhead.

Furthermore, we give the memory overhead of each deep learning model as depicted in Figure 16. Because the CNN-based model was implemented using Keras [33] and it is more complex than the the SC-LSTM based and the attention-based models, its memory overhead is much larger than the others. We believe that the attention-based model is suitable for devices with less memory.

## 6. Discussion

In this study, we proposed an attention-based detection model. It has approximately 12% and 5% higher accuracy than the CNN-based and SC-LSTM-based models. However, the attention-based model requires a longer training time than the CNN-based and SC-LSTM-based models since it must extract a data subsequece of length *k* as shown in Figure 13. However, when we detect malware with anti-virus software, the training time is not important because we can use the pre-trained neural network by the attention-based model. Because the test time of the attention-based model is less than 1 ms per file as shown in Figure 14, it is suitable for use in real anti-virus software.

## 7. Conclusions

In this study, we proposed an attention-based feature extraction method, and verified the performance of the deep learning-based malicious file detection model utilizing this method. The deep learning-based malicious file detection model using an attention-based feature extraction method yields an accuracy that is approximately 12% and 5% higher than those of a CNN-based model and SC-LSTM model, respectively.

In addition, the attention-based feature extraction method allows malicious code analysts to only analyze parts of malicious code based on the features extracted by the attention-based feature extraction method, rather than analyzing the entire malicious code. This is expected to considerably reduce the efforts required by malicious code analysts. 

## Figures and Tables

**Table 1:** API system call list.

API Num	API System Call	Category
1	CreateProcess	1
2	ExitProcess	1
3	TerminateProcess	1
4	OpenProcess	1
5	SearchProcess	1
6	ProcessDEPPolicy	1
7	InformationProcess	1
8	CreateLocalThread	2
9	ExitThread	2
10	TerminateThread	2
…	…	…

**Table 2:** Experimental environments.

Name	Specification
OS	Ubuntu 14.04 (64 bit)
CPU	Intel i7-7700 (4.2 GHz)
RAM	32 GB
GPU	GTX 1060
Cuda	8.0

**Table 3:** Types of malware.

Malware Type	Number
Trojan	646
Win32	281
Backdoor	23
Worm	25
Dropper	6
Malware	4
Virus	4
Total	1000

**Table 4:** Confusion matrix.

-	Malware	Benign File
Predicted Malware	TP	FP
Predicted Benign File	FN	TN

**Table 5:** Accuracy results.

Model	Seq Length	TP	FP	FN	TN	Accuracy
CNN	200	115	28	23	129	82.71
CNN	400	106	14	32	143	84.40
CNN	600	107	9	31	148	86.44
CNN	800	107	13	31	144	85.08
SC-LSTM	200	121	11	17	146	90.50
SC-LSTM	400	123	12	15	145	90.84
SC-LSTM	600	123	7	15	150	92.54
SC-LSTM	800	125	15	13	142	90.50
Attention	200	130	5	8	152	95.59
Attention	400	131	5	7	152	95.93
Attention	600	132	5	6	152	96.27
Attention	800	125	15	13	142	90.50
